# Adoption of improved soybean and gender differential productivity and revenue impacts: Evidence from Nigeria

**DOI:** 10.1002/fes3.385

**Published:** 2022-05-06

**Authors:** Amadu Y. Kamara, Oyakhilomen Oyinbo, Julius Manda, Lucy S. Kamsang, Nkeki Kamai

**Affiliations:** ^1^ 54715 Department of Agricultural Economics Obafemi Awolowo University Ife Nigeria; ^2^ 58989 Department of Agricultural Economics Ahmadu Bello University Zaria Nigeria; ^3^ 105528 International Institute of Tropical Agriculture Arusha Tanzania; ^4^ Department of Agricultural Economics Bayero University Kano Nigeria; ^5^ 105528 International Institute of Tropical Agriculture Ibadan Nigeria

**Keywords:** adoption, endogenous switching regression, improved soybean varieties, net revenue, yield

## Abstract

Despite the considerable soybean varietal improvement and dissemination efforts in Nigeria and other parts of Sub‐Saharan Africa, empirical evidence on farm‐level yield and revenue impacts of improved soybean varieties (ISVs) from a gender perspective are limited. In this paper, we analyze the impact of the adoption of ISVs on soybean yield and net revenue, and the associated gender differential effects in northern Nigeria. We use the endogenous and exogenous switching treatment effects regression frameworks to estimate the impacts. We find that the adoption of ISVs significantly increased soybean yield and net revenue of the soybean‐producing households by 26% and 32%, respectively. In addition, we find that the gender gap in yield between male and female‐headed soybean‐producing households was small, with a yield gap of about 1%. However, we find a substantial gender gap in soybean net revenue, as the net revenue of female‐headed households was lower by about 20%, as compared to male‐headed households. Overall, our findings show that policymakers and their development partners can leverage varietal improvement to boost the yields of both male‐ and female‐headed households. However, closing the gender gap in crop income necessitates reducing the disparity in market linkages so that the female farmers can equally have better market access.

## Graphical abstract

Field surveys were conducted to evaluate the impact of the adoption of improved soybean varieties in Northern Nigeria and the gender differences in impact. Results show that the adoption of improved soybean varieties by both male and female farmers significantly increased yield and net revenue. While there were no differences between male and female farmers in terms of yield, male farmers had significantly higher income than their female counterpart.

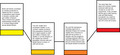

## 1 INTRODUCTION

Soybean (Glycine Max) is an important cash crop for rural households in the Nigerian Savannas and other parts of Sub‐Saharan Africa (SSA) partly due to its rising industrial demand (Mahama et al., 2020; Ugbabe et al., 2017). It is promoted among smallholders not only for food and cash but for improving soil fertility in cereal‐dominated cropping systems through biological nitrogen fixation (Ulzen et al., 2018; Vanlauwe et al., 2019). In addition, it is beneficial in reducing the infestation of parasitic weeds, in cereal fields (Franke et al., 2004; Kamara et al., 2008). While Nigeria is the second‐largest producer of soybean in Africa after South Africa, (FAOSTAT, 2021), the yield is on average <1 ton/ha, which is below the potential yield of over 3 tons/ha (Ronner et al., 2016). Biophysical constraints, such as pest and diseases, drought, poor soil fertility, high pod shattering, poor agronomic practices, and market‐related constraints contribute to the low soybean yields (Kamara et al., 2014; Khojely et al., 2018).

In response to these challenges, the International Institute of Tropical Agriculture (IITA) has for many decades, worked in close collaboration with national partners to develop improved soybean varieties (ISVs) along with complementary agronomic practices (Dugje et al., 2006; Vanlauwe et al., 2019). The improved varieties have important technological traits, such as high yield, drought tolerance, resistance to pests and diseases, low pod shattering, high seed viability, and early maturity. In particular, the climate‐resilience traits (e.g., drought tolerance, early maturity) of most of the varieties has enabled the spread of soybean production from the Guinea Savannas of Nigeria to drier agro‐ecologies, such as the Sudan Savanna (Ugbabe et al., 2017). Several interventions have been implemented to disseminate the ISVs and associated management practices among smallholders in Nigeria (Amaza, 2016; Bamire et al., 2010). Because soybean is a crop cultivated and processed by women, these interventions all have gender mainstreaming activities to help reduce inequalities in soybean production and household welfare between male and women farmers (Amaza, 2016).

Recent studies in economic literature have documented the adverse effects of gender inequality on broader economic growth (Burke & Jayne, 2021; Glazebrook et al., 2020; Wodon & De La Brière, 2018). At the household level, empirical evidence of differences in productivity as a result of gender disparity has been documented by several studies (e.g., Burke & Jayne, 2021; Diiro et al., 2018; Mugisha et al., 2019; Obisesan, 2021; Tambo et al., 2021). Tambo et al. (2021) for example reported that while participation in plant clinics resulted in productivity growth for maize farmers in Zambia, the effect was disproportionately greater for male farmers. Similarly, Mugisha et al. (2019) reported that female plot managers had less groundnut yield than their male counterparts in Uganda due to some structural disadvantages they face in comparison to their male counterparts.

There is a large body of literature on the adoption, productivity, and welfare impacts of improved agricultural technologies in Nigeria and SSA in general (e.g., Abdoulaye et al., 2018; Amare et al., 2012; Asfaw et al., 2012; Jaleta et al., 2018; Kassie et al., 2011, 2013, 2018; Khonje et al., 2015; Manda et al., 2019, 2020; Shiferaw et al., 2014; Wossen et al., 2019). However, the focus of the previous studies is largely on maize, cassava, cowpea, groundnut, and pigeon pea. In Nigeria, empirical findings on impacts of improved technologies have been documented for maize (e.g., Abdoulaye et al., 2018; Oyinbo et al., 2019), for cowpea (e.g., Alene et al., 2006; Manda et al., 2019, 2020) and for cassava (e.g., Awotide et al., 2015; Wossen et al., 2019). Despite the considerable soybean varietal improvement and dissemination efforts in Nigeria, there is thin rigorous evidence on yield and revenue impacts of ISVs, especially in the North‐eastern Nigeria where these varieties have been promoted over the years. In addition, while women are actively involved in soybean production in our study setting, no empirical study has evaluated the gender differential effects of soybean production in the nation. Even in SSA in general, studies on the impacts of ISVs are limited, except for Tufa et al. (2019) in Malawi to our knowledge.

In this paper, we analyze the *ex‐post* impacts of the adoption of ISVs on soybean yield and net revenue in the North‐eastern region of Nigeria using plot‐level data. Our focus on this region is particularly of policy relevance because the region is plagued with several development challenges, including the *Boko haram* armed insurgency, which makes it in dire need of yield‐enhancing technologies that can deliver welfare benefits to smallholders. Our contributions to the literature are two‐fold. First, we provide rigorous evidence on the productivity and revenue impacts of improved soybean, a crop that has received limited attention in the agricultural technology adoption and impact literature. We estimate the impact of the adoption of ISVs on soybean yield and net revenue using the endogenous switching regression model to account for the potential endogeneity of adoption. Our paper builds on Sanginga et al. (1999) who attempted to estimate the social impact of soybean, but in a rather qualitative manner, with a small sample size, in a different region (north‐central region of Nigeria). Second, we empirically estimate the impact of gender on soybean yield and net revenue using the exogenous switching treatment effect regression. This allows us to provide useful insights on yield and net revenue impacts of agriculture‐related technologies from the perspective of gender, which has not received much attention in most of the previous impact studies highlighted above, despite the crucial role of gender in sustainable development.

The rest of the paper is organized as follows: In the next section, we briefly describe the soybean interventions in north‐eastern Nigeria. In Section 3, we describe the study area and the data employed in the paper. In Section 4, we describe the conceptual framework and estimation strategy of the paper. In Section 5, we discuss the results and conclude the paper in Section 6.

## 2 SOYBEAN INTERVENTIONS IN NORTH‐EASTERN NIGERIA

Soybean cultivation before 2004 in north‐eastern Nigeria was very limited, particularly in Borno State (Amaza et al., 2007). Donor‐funded projects led by IITA that promoted ISVs with the collaboration of national partners include the Promoting Sustainable Agriculture in Borno State (PROSAB) project (2004–2009), the Tropical Legumes II (TL‐II) project (2007–2015), and the N2Africa project (2014–2018). The PROSAB project first introduced ISVs and other agronomic management practices and provided linkages to input and output markets. From 2004, the project largely promoted the soybean variety, TGX 1448‐12E, which is late maturing and relatively low yielding due to its susceptibility to soybean rust disease and delayed flowering as a result of photosensitivity. Due to the constraints associated with TGX1448‐12E, the TL‐II project supported the dissemination of new varieties to address these constraints. The varieties introduced by the TL‐II project that are early‐maturing, include TGX 1951‐3F, TGX 1955‐4F, and TGX 1904‐6F, and an extra‐early maturing variety, TGX 1835‐10F. They are all high‐yielding, drought‐tolerant, and resistant to pests and disease (Abate et al., 2012). The N2Africa project also promoted the use of varieties promoted by the TL‐II project. In addition, the N2Africa project promoted the use of additional inputs, such as rhizobium inoculants and phosphorus fertilizers as complementary technologies that can substantially boost soybean yield (Amaza et al., 2007). In general, the projects strongly considered gender mainstreaming and ensured that male and female farmers were equally targeted. This was aimed at reducing economic and social inequalities that exist between male and female farmers.

## 3 STUDY AREA AND DATA

Our study was carried out in Borno State, located in North‐eastern Nigeria. The state has four agro‐ecological zones, including southern and northern Guinea savannas in the south, Sudan savanna in the central parts and Sahel savanna in the north. Our study covered the three major soybean‐producing areas in the state, which are Hawul, Kwaya Kusar, and Biu Local Government Areas (LGAs)—an LGA is the smallest administrative unit in the state. We used a two‐stage sampling procedure to select the soybean‐producing households in the three LGAs. In the first stage, we used a probability proportional to size sampling to randomly select 14 communities from the list of communities in Hawul and Biu LGAs, respectively, and 12 communities from Kwaya Kusar LGA, which gave a sample of 40 communities. In the second stage, a sampling frame of soybean‐producing households was constructed for the 40 communities. In each of the communities, the soybean‐producing households were stratified by gender of the household heads and ten male‐headed households (MHHs) and ten female‐headed households (FHHs)^1^ were randomly selected from the list of soybean‐producing households, which results in a total sample of 800 households with subsamples of 400 MHHs and 400 FHHs (see Table A1 in the Appendix).

Our study relied on data from a survey that was implemented in October–November 2017 under the IITA‐led N2Africa Borno project. The data were collected at both the household and plot levels from the sample of 800 households who cultivated 1094 soybean plots (566 plots for MHHs and 528 plots for FHHs). The survey instrument was a structured quantitative questionnaire. It had modules on household demographic characteristics, land ownership, social capital, extension, credit, assets, access to institutional services, adoption of ISVs, plot‐level soybean production inputs and costs, and the associated output and prices. We implemented the survey with three survey teams, comprising of six enumerators and two supervisors in each team led by research fellows at IITA, Kano station, Nigeria. The questionnaire was administered to the farmers by the enumerators with the help of computer‐assisted personal interview (CAPI) using open data kit software (ODK) to improve the quality of data collection.

## 4 CONCEPTUAL FRAMEWORK AND ESTIMATION STRATEGY

### 4.1 Conceptual framework

Based on the utility maximization theory and consistent with empirical literature (Abdoulaye et al., 2018; Khonje et al., 2015; Manda et al., 2019; Tufa et al., 2019), we expect that farmers’ adoption of ISVs would occur when the expected utility associated with adoption, $U_{\mathrm{adopt}}$ is greater than that associated with non‐adoption, $U_{\mathrm{non}‐\mathrm{adopt}}$. In this sense, if we assume that the latent variable $T^{*}\left(U_{\mathrm{adopt}}‐U_{\mathrm{non}‐\mathrm{adopt}}\right)$ represents the utility (net benefits) from adoption, $T^{*}>0$ implies that a farmer will adopt an ISV given that the $U_{\mathrm{adopt}}>U_{\mathrm{non}‐\mathrm{adopt}}.$ However, $T^{*}$ cannot be observed, and we express it as a latent variable, which is a function of observable covariates:

(1) $$\begin{array}{c} T^{*}=\beta X_{i}+\mu_{i}\;\text{with}\;T_{i}=\left\{\begin{array}{cc} 1 & \text{if}\;Y^{*}>0\\ 0 & \text{otherwise} \end{array}\right. \end{array}$$

Where $T_{i}$ is a binary indicator variable that takes a value of 1 if a farmer is an adopter and 0 otherwise. We defined an adopter as a farmer who cultivated any of the ISVs, including the early and extra‐early maturing varieties – TGX1904‐6F, TGX1835‐10F, TGX 1951‐3F, and TGX 1955‐4F on any of his/her plots in the 2017 cropping season.^2^ A non‐adopter of ISV is any farmer who cultivated older varieties that are late maturing, low yielding and susceptible to rust disease (e.g., TGX1448‐12E) in the 2017 cropping season. $X_{i}$ is a vector of observable household, farm, and institutional characteristics, β is a vector of parameters associated with $X_{i}$ and $\mu_{i}$ is the error term. The adoption of ISVs is expected to improve soybean yield and net revenue. This assertion is based on the impact pathway of agricultural research for development. Research on germplasm improvement generates improved soybean varieties that are drought‐tolerant, resistant to pests and diseases, have low pod‐shattering, etc. to mitigate biotic and abiotic stresses. When farmers adopt these new varieties based on their perception of certain desirable traits, they minimize losses due to productivity shocks and increase yield. This will lead to an increase in market output thereby raising the income of farmers (Alwang et al., 2019). Hence, the implicit relationship between the adoption of ISVs and the two outcome variables is as follows:

(2) $$Y_{i}=\gamma_{0}+\;\gamma_{1}T_{i}+\gamma_{2}X_{i}+\varepsilon_{i}$$

Where $Y_{i}$ represents the outcome variables—soybean yield (kg/ha) and net revenue (Nigerian Naira‐NGN/ha) for an $i^{\mathrm{th}}$ household. Net revenue is the soybean revenue (value of output) less the variable costs of production per ha.

Equation (2) expresses the adoption of ISVs as an exogenous variable, which only holds when farmers are randomly assigned to treatment (adopter) or control (non‐adopter) groups. Given that the decision to adopt may be due to observable and unobservable characteristics of farmers, adoption is not random as the group of farmers that adopt may be systematically different from the non‐adopters, which raises concern about self‐selection bias. In addition, when unobservable factors (e.g., management abilities, entrepreneurial skills, and motivation) affect both the technology choice and the outcomes of interest, the error terms of Equations (1) and (2) are correlated. Estimation of Equation (2) without controlling for the unobserved heterogeneity will yield biased and inconsistent estimates of $\gamma_{1}$ (Angrist & Pischke, 2008).

### 4.2 Endogenous switching regression (ESR)

To account for both observable and unobservable sources of heterogeneity, we estimated the yield and revenue impacts of the adoption of improved soybean varieties using the endogenous switching regression (ESR) model (Lee, 1978; Shiferaw et al., 2014), as implemented in recent empirical impact studies (e.g., Abdoulaye et al., 2018; Abdulai & Huffman, 2014; Di Falco et al., 2011; Jaleta et al., 2018; Manda et al., 2019). However, the estimation of ESR requires an instrumental variable—a variable that is strongly correlated with the decision to adopt but does not directly affect yield and net revenue. In this way, the indirect influence of the instrument on the outcomes only emerges through its effect on ISV adoption. The selection instruments considered in this study are distance to an agricultural extension office and access to varietal information from different sources. These are plausible instruments as we expect that farmers who live close to extension service providers and have access to information on ISVs from multiple information sources are more likely to have better access to information on ISVs and related technologies, which can lead to better‐informed decisions on technology choice. In this regard, relaxing information constraints on the availability, technical know‐how and expected benefits of technologies can play a crucial role in the adoption behavior of farmers (Adegbola & Gardebroek, 2007). The use of distance to an agricultural extension office and access to varietal information as instruments is quite common in the empirical impact evaluation literature (e.g., Abdoulaye et al., 2018; Khojely et al., 2018; Manda et al., 2019; Shiferaw et al., 2014). Following Di Falco et al. (2011), we performed a falsification test to ascertain the validity of the instrument. Table A2 and A3 in the Appendix show that the instruments (access to varietal information and distance to extension office) are valid, as they are jointly correlated with the adoption decisions of farmers at the 1% significance level, but not correlated with yield and net revenue.

While we have carefully motivated the choice of our instruments from theory and empirical applications in previous studies, we acknowledge that the exogeneity of our instruments is not incontestable. For example, it may be contested that wealthier households may be more likely to reside in areas closer to an LGA’s headquarter, which is where an agricultural extension office is often located, and they may be more likely to have access to information on ISVs from multiple information sources. Thus, our results should be interpreted with care.^3^

The econometric framework for the ESR model follows two stages and we use an efficient estimation method, the full information maximum likelihood (FIML) to estimate the model (Lokshin & Sajaia, 2004). In the first stage, the probability of adoption is estimated using a probit regression expressed in Equation (1), that is, the estimation of the selection equation. In the second stage, the relationship between the outcomes of interest (yield and net revenue) and the household, farm and institutional characteristics are estimated using an OLS regression with selectivity correction under two regimes, conditional on adoption. The two regimes are expressed with outcome Equations (3a) and (3b)

(3a) $$\begin{array}{c} \mathrm{Regime}\;1\;\left(\mathrm{adopters}\right):y_{1i}=\gamma_{1}X_{1i}+\;\epsilon_{1i}\;\;\;\;\mathrm{if}\;T_{i}=1 \end{array}$$

(3b) $$\begin{array}{c} \mathrm{Regime}\;2\;\left(\mathrm{non}‐\mathrm{adopters}\right):y_{2i}=\gamma_{2}X_{2i}+\;\epsilon_{2i}\;\;\;\;\mathrm{if}\;T_{i}=0 \end{array}$$

Where $y_{1i}$ and $y_{2i}$ represents outcomes for the adopters and non‐adopters of ISVs, respectively. $X_{i}$ is a vector of observable household, farm and institutional characteristics, γ is a vector of parameters associated with $X_{i}$ and $\epsilon_{i}$ the error term. The error terms in Equations (1) and (2) are assumed to have a trivariate normal distribution, with zero mean and non‐singular covariance matrix expressed as:

(4) $$cov\;\left(\mu_{i},\epsilon_{1i},\epsilon_{2i}\right)=\left(\begin{array}{ccc} \sigma_{1}^{2} & \sigma_{12} & \sigma_{1\mu }\\ \sigma_{21} & \sigma_{2}^{2} & \sigma_{2\mu }\\ \sigma_{\mu 1} & \sigma_{\mu 2} & \sigma_{\mu }^{2} \end{array}\right)$$

Where $\sigma_{\mu }^{2}$ is the variance of the error term in Equation (1), $\sigma_{1}^{2}$ and $\sigma_{2}^{2}$ are the variances of the error terms in Equations (3a) and (3b) respectively, $\sigma_{1\mu }$ is the covariance of $\mu_{i}$ and $\epsilon_{1i}$, $\sigma_{2\mu }$ is the covariance of $\mu_{i}$ and $\epsilon_{2i}$. It is plausible to assume that $\sigma_{\mu }^{2}$ equals to one since the β coefficients in Equation (1) are estimable up to a scale factor (Maddala, 1986). Given that the outcomes of interest, $y_{1i}$ and $y_{2i}$ are not observed simultaneously, the covariance between $\epsilon_{1i}$ and $\epsilon_{2i}$ is not defined (Maddala, 1986). The expected values of $\epsilon_{1i}$ and $\epsilon_{2i}$ conditional on sample selection is non‐zero because $\mu_{i}$ in Equation (1) is correlated with $\epsilon_{1i}$ and $\epsilon_{2i}$ in Equations (3a) and (3b) respectively. The expected values of the error terms in Equations (3a) and (3b) can be expressed as follows:

(5a) $$E\left(\epsilon_{1i}|y_{1i}=1\right)=\sigma_{1\mu }\frac{\emptyset (\beta X_{i})}{\Phi (\beta X_{i})}=\sigma_{1\mu }\lambda_{1i}$$

(5b) $$E\left(\epsilon_{2i}|y_{2i}=1\right)=\sigma_{2\mu }\frac{\emptyset (\beta X_{i})}{\Phi (\beta X_{i})}=\sigma_{2\mu }\lambda_{2i}$$

where ∅(.) is the standard normal probability density function and Φ(.) is the standard normal cumulative density function. $\lambda_{1i}$ and $\lambda_{2i}$ are the inverse Mills ratios (IMR) estimated from the selection Equation (1) and then included in the outcome Equations (3a) and (3b), respectively to correct for selection bias. Using the ESR framework expressed above, the average treatment effect of the treated (ATT) can be obtained by comparing the expected values of the outcomes of adopters in actual and counterfactual scenarios. To this end, the expected values of the outcomes of adopters and non‐adopters of ISVs in actual and counterfactual scenarios are expressed as follows:

Adopters with the adoption of ISVs (actual scenario)

(6a) $$E\left(y_{1i}|X_{1i}=1\right)=\gamma_{1}X_{1i}+\sigma_{1\mu }\lambda_{1i}$$

Adopters without adoption of ISVs (counterfactual scenario)

(6b) $$E\left(y_{2i}|X_{1i}=1\right)=\gamma_{2}X_{1i}+\sigma_{2\mu }\lambda_{1i}$$

The ATT for adopters is computed as the difference between (6a) and (6b), which is the impact of the adoption of ISVs on the outcomes of interest for the adopters.

(7) $$\mathrm{ATT}=E\left(y_{1i}|X_{1i}=1\right)‐E\left(y_{2i}|X_{1i}=1\right)=X_{1i}\left(\gamma_{1}‐\gamma_{2}\right)+\lambda_{1i}\left(\sigma_{1\mu }‐\sigma_{2\mu }\right)$$

As robustness checks, we estimate the impact of ISVs on yield and net revenue using the augmented inverse‐probability weighting (AIPW). The AIPW is a doubly robust estimator which provides efficient estimates by allowing the modeling of the outcome and the treatment equations while requiring that only one of the two models be correctly specified to consistently estimate the impact (Wooldridge, 2010). In the interest of brevity, we do not describe these methods. For a detailed description, see Imbens and Wooldridge (2009) and Wooldridge (2010).

### 4.3 Exogenous switching treatment effect regression

The exogenous switching treatment effect regression (ESTER) is used in this study to examine the gender gaps in soybean yield and net revenue associated with the adoption of ISVs. A more intuitive approach would be to simply employ a pooled regression with a dichotomous gender variable, that is, a dummy variable that disaggregates MHHs and FHHs. The limitation of this approach lies in the fact that while the inclusion of a gender dummy variable in a pooled regression will estimate the intercept effect (i.e., a homogenous shift in slope), it will not consider the interactions between gender and other explanatory variables of the model (Kassie et al., 2015; Muricho et al., 2020). The latter implies that gender only has an intercept effect or a parallel shift effect, which is constant regardless of the values taken by other covariates that determine soybean yield and net revenue.

The use of ESTER framework allows us to address such interactions between gender and other explanatory variables by estimating two separate equations for MHHs and FHHs as follows:

(8a) $$y_{m}=x_{m}\gamma_{m}+v_{m}\;\text{if}\;g=1$$

(8b) $$y_{f}=x_{f}\gamma_{f}+v_{f}\;\text{if}\;g=0$$

In Equations (8a and 8b), m and f represent MHHs and FHHs, respectively, while g is the dichotomous choice variable, which is 1 if the head of the household is a male and 0 if the head is a woman. The variables x and y in both expressions represent the vectors of household characteristics and yield and net revenue, respectively. The parametric coefficients $\gamma_{m}$ and $\gamma_{f}$ capture how MHH and FHH soybean yield and net revenue react to the vector of household characteristics while $\nu_{m}$ and $\nu_{f}$ are the error terms, with both having the properties of constant variance and zero means. But the model specified in Equation ((8a), (8b)) may not allow us to directly examine the role of gender in yield and net revenue for MHHs and FHHs because of differences in their household characteristics. To be able to do this, we estimate the counterfactual of the yield and net revenue levels of each group. This counterfactual value is what the outcomes in yield and net revenue of FHHs would be if the returns on their characteristics had been the same as the returns on the MHHs characteristics and vice versa. Following Kassie et al. (2015) and Carter and Milon (2005), we computed the actual and counterfactual soybean yield and net revenue of MHHs and FHHs as follows;

(9a) $$E\left(y_{m}|g=1\right)=x_{m}\gamma_{m}$$

(9b) $$E\left(y_{f}|g=0\right)=x_{f}\gamma_{f}$$

(9c) $$E\left(y_{f}|g=1\right)=x_{m}\gamma_{f}$$

(9d) $$E\left(y_{m}|g=0\right)=x_{f}\gamma_{m}$$

Equations (9a) and (9b) represent the soybean yield and net revenue for MHHs and FHHs observed in the sample respectively while Equations (9c) and (9d) represent the expected yield and net revenue of MHHs and FHHs, respectively. We decompose the gap in yield and net revenue into the portion of the gender gap that is caused by differences in the levels or quantity of observable resources between both groups (level effect), and the portion of the gender gap explained by differences in the returns to these resources (returns effect). The returns effect of gender on the yield gap and net revenue is measured under the condition that the characteristics of MHHs’ have the same returns as FHHs’ characteristics. The returns effect of gender on MHHs yield and net revenue ($M_{p}$) would be given as the difference between Equations (9a) and (9c):

(10) $$R_{M}=E\left(y_{m}|g=1\right)‐E\left(y_{f}|g=1\right)=x_{m}\left(\gamma_{m}‐\gamma_{f}\right)$$

Similarly, the effect of gender on FHHs yield and net revenue ($R_{F}$) would be given as the difference between Equations (9d) and (9b)

(11) $$R_{F}=E\left(y_{m}|g=0\right)‐E\left(y_{f}|g=0\right)=x_{f}\left(\gamma_{m}‐\gamma_{f}\right)$$

Equations (10) is the average treatment effect on the treated while Equation (11) is the average treatment effect on the untreated.

The gap in the outcomes due to differences in the level of observable characteristics for MHHs (level effect) is given as the difference between equations (9a) and (9d)

(12) $$L_{M}=E\left(y_{m}|g=1\right)‐E\left(y_{m}|g=0\right)=\gamma_{m}\left(x_{m}‐x_{f}\right)$$

The level effect for FHHs is given as the difference between Equation (9c) and (9b)

(13) $$L_{F}=E\left(y_{f}|g=1\right)‐E\left(y_{f}|g=0\right)=\gamma_{m}\left(x_{m}‐x_{f}\right)$$

To test the robustness of the ESTER results, we estimate the Oaxaca‐Blinder (OB) decomposition model, and the results are presented in Table A6 and A7 in the appendix. For a detailed discussion of the OB decomposition model, see Oaxaca (1973), Blinder (1973) and empirical applications in agricultural economics studies (e.g., Aguilar et al., 2015; Marenya et al., 2017; Mugisha et al., 2019; Muricho et al., 2020).

## 5 RESULTS AND DISCUSSION

### 5.1 Summary Statistics

Tables 1 and 2 show the household, farm and institutional characteristics of the soybean‐producing households by adoption and by gender, respectively. These characteristics are selected based on previous empirical studies in the adoption and impact literature (e.g., Jaleta et al., 2018; Kassie et al., 2018; Khonje et al., 2015; Manda et al., 2020; Nguezet et al., 2020; Shiferaw et al., 2014; Tufa et al., 2019). On average, the adopters of ISVs varieties had a significantly higher education, had better access to market information, owned more mobile phones, and had a lower distance to seed market and extension service providers (Table 1). On the other hand, in terms of statistical significance non‐adopters had a larger household size than adopters. In terms of the outcome variables, the adopters had a significantly higher soybean yield and net revenue than non‐adopters, with both results being statistically significant. In addition, the adopters from both MHHs and FHHs had a significantly higher yield and net revenue than their counterparts who are non‐adopters.

**TABLE 1:** Summary statistics of farm‐households by adoption status

Variable	Full sample	Adopters	Non‐adopters	Difference
Dependent variables				
Soybean yield (kg/ha)	2312.187	2452.182	1897.275	554.91 (43.05)***
MHHs soybean yield (kg/ha)	2325.345	2451.497	1897.995	553.5 (61.01)***
FHHs soybean yield (kg/ha)	2298.082	2452.967	1896.644	556.32 (61.15)***
Soybean net revenue (NGN/ha)	194,142.7	207,102.3	155,733.5	51,368.83 (5131.76)***
MHHs net revenue (NGN/ha)	214,079.4	226,599.3	171,667	54,932.26 (7871.99)***
FHHs net revenue (NGN/ha)	172,771.2	184,739.6	141,751	42,988.65 (6040.15)***
Explanatory variables				
Education of HH head (years)	1.93	2.05	1.63	0.42 (3.20)***
Household size (no. of HH members)	8.14	7.74	9.19	−1.45 (3.80)***
Membership of association (yes = 1)	0.44	0.43	0.44	−0.01 (0.15)
Access to credit (yes = 1)	0.07	0.06	0.07	0.01 (0.50)
Access to varietal information (yes = 1)	0. 71	0.79	0.50	0.28 (8.39)***
Years HH is resident in community	29.39	33.62	18.33	15.29 (12.88)***
Access to off‐farm income (yes = 1)	0.64	0.67	0.55	0.11 (3.00)***
Value of HH assets per capita (NGN)	13,154.01	13,605.59	11,970.9	1634.69 (0.8)
Value of farm implements (NGN)	31,827.13	33,208.97	28,206.81	5002.163 (7.04)***
Mobile phone ownership (yes = 1)	0.90	0.92	0.84	0.08 (3.4)***
Transport asset ownership (yes = 1)	0.50	0.53	0.43	0.09 (2.3)**
Tropical livestock units	0.91	0.9	0.93	−0.02 (0.15)
Total land cultivated (ha)	2.86	3.04	2.34	0.7 (0.14)***
Use of SSP fertilizer (yes = 1)	0.69	0.74	0.55	0.19 (0.03)**
Use of herbicide (yes = 1)	0.16	0.16	0.17	−0.01 (0.03)
Distance to output market (km)	3.80	3.98	3.34	0.64 (1.6)
Distance to seed market (km)	5.58	5.05	6.97	1.93 (3.5)***
Distance to primary school	2.43	2.62	1.95	0.67 (1.78)*
Distance to extension service (km)	7.06	6.15	9.45	3.3 (4.25)***
Constrained by low soil fertility^a^	4.57	4.63	4.44	0.19 (0.6)
Constrained by high cost of inputs^a^	5.41	5.27	5.76	0.49 (1.65)
Constrained by pests and diseases^a^	4.65	4.67	4.58	0.09 (0.3)
Biu LGA (yes = 1)	0.33	0.32	0.35	0.03 (0.85)
Kwaya Kusar LGA (yes = 1)	0.27	0.22	0.41	0.19 (5.55)***

Notes

Standard error in parentheses, ***, **, and * denote significance at 1%, 5%, and 10% levels respectively.

^a^ Perceived severity of constraints on a scale of 10, from zero (not constrained) to 10 (severely constrained), NGN: 305 NGN (Nigerian Naira) is equivalent to 1 USD at the survey time.^4^

**TABLE 2:** Summary statistics of farm‐households by gender

Variable	Full sample	MHHs	FHHs	Difference
Adopt improved soybean varieties (yes = 1)	0.75	0.77	0.72	0.05 (0.03)
Dependent variables				
Soybean yield (kg/ha)	2312.19	2325.35	2298.08	27.26 (40.16)
Soybean net revenue (NGN/ha)	194,142.70	214,079.40	172,771.20	41,308.21 (4489.76)***
Explanatory variables				
Education of HH head (years)	1.93	1.88	1.99	0.1 (0.85)
Household size (number of HH members)	8.14	8.17	8.12	0.05 (0.15)
Membership of association (yes = 1)	0.44	0.52	0.35	0.17 (4.75)***
Access to credit (yes = 1)	0.07	0.06	0.07	0.01 (0.3)
Years HH is resident in community	29.39	28.95	29.83	0.88 (0.73)
Access to varietal information (yes = 1)	0.71	0.70	0.72	0.02 (0.55)
Access to off‐farm income (yes = 1)	0.64	0.66	0.61	0.05 (1.45)
Value of HH assets per capita (NGN)	13,154.01	13,638.97	12,669.04	969.93 (0.55)
Value of farming implements (NGN)	31,827.13	31,824.97	31,829.28	4.30(6.6E−3)
Mobile phone ownership (yes = 1)	0.90	0.96	0.84	0.11 (5.25)***
Tropical livestock units	0.91	0.84	0.98	0.14 (0.8)
Total land cultivated (ha)	2.86	3.22	2.48	0.75 (0.12)***
Use of SSP fertilizer (yes = 1)	0.69	0.70	0.68	0.02 (0.03)***
Use of herbicide (yes = 1)	0.16	0.17	0.15	0.02 (0.02)
Distance to output market (km)	3.80	3.80	3.81	0.01(0.01)
Distance to seed market (km)	5.58	5.66	5.50	0.17(0.35)
Distance to extension service (km)	7.06	7.13	6.99	0.14(0.2)
Constrained by low soil fertility^a^	4.57	4.67	4.48	0.2(0.7)
Constrained by high cost of inputs^a^	5.41	5.33	5.49	0.16(0.6)
Constrained by pests and diseases^a^	4.65	4.65	4.64	0.01(0.05)
Biu LGA (yes = 1)	0.33	0.33	0.32	0.01(0.15)
Kwaya Kusar LGA (yes = 1)	0.27	0.25	0.29	0.04(1.45)

Note

Standard error in parentheses, *** and * denote significance at 1% and 10% levels, respectively.

^a^ Perceived severity of constraints on a scale of 10, from zero (not constrained) to 10 (severely constrained), NGN: 305 NGN (Nigerian Naira) is equivalent to 1 USD at the survey time.

Table 2 shows that 75% and 70% of the MHHs and FHHs, respectively, adopted ISVs and the mean difference is statistically significant at the 10% level. While there is no significant difference in yield between the MHHs and FHHs, the MHHs had about 22% higher net revenue than FHHs. In addition, the MHHs had a higher membership in associations, owned more mobile phones, and cultivated more land than FHHs, with all these differences being statistically significant. Inferring causality from the mean differences in yield and net revenue of adopters and non‐adopters would however be biased because adopters are systematically different from non‐adopters in most of the observable characteristics.

### 5.2 ESR estimates of the yield and net revenue impacts of ISVs

#### 5.2.1 ESR estimates of determinants of ISVs adoption

The full information maximum likelihood estimates of the determinants of adoption of ISVs (selection equations) in the ESR model are presented in Column (1) in Tables 3 and 4, respectively The results from the selection equation show that the drivers of the adoption of ISVs include, education of HH head, access to credit, access to off‐farm income, household size, association membership, years household head is resident in the community, size of land cultivated, use of herbicides, distance to output market, distance to seed market, distance to extension service providers, and access to varietal information. These results are consistent with the findings of previous studies on agricultural technology adoption in SSA (Asfaw et al., 2012; Kassie et al., 2011; Manda et al., 2019; Wossen et al., 2019).

**TABLE 3:** Full information maximum likelihood of endogenous switching regression—Soybean yield

Variable	Selection equation		Outcome equations			
			Adopters		Non‐adopters	
	Coefficient	std. err.	Coefficient	std. err.	Coefficient	std. err.
Male headed household	0.16	1.46	−8.59	47.21	−2.71	56.94
Education of HH head	0.42***	4.35	−50.60	40.22	−37.96	57.47
Household size	−0.06***	4.96	13.57***	5.35	−2.44	6.07
Membership of association	0.22*	1.94	−74.64	48.68	−75.17	64.63
Access to credit	0.56***	2.48	126.75	93.53	−105.13	124.26
Years HH is resident in community	0.95***	12.93	0.68	44.59	−206.85***	76.63
Access to off‐farm income	0.34***	3.10	104.13**	48.36	105.54	59.37
Value of HH assets	0.00	0.12	31.32**	16.01	−13.29	20.51
Value of farming implements	1.13***	6.76	92.70	81.59	205.86*	109.08
Mobile phone	0.21	1.23	174.40**	88.29	−96.64	82.82
Tropical livestock unit	0.09	0.87	22.74	40.39	−3.23	54.42
Total land cultivated	0.27***	3.46	−13.52	34.78	71.18*	42.85
Use of SSP	0.68	5.71	30.11	55.65	143.69*	75.54
Use of herbicide	−0.54***	3.61	35.08	64.86	−112.37	86.91
Constrained by low soil fertility	0.01	0.88	11.74**	5.82	4.82	7.06
Constrained by high cost of inputs	−0.02	1.50	−11.19*	6.13	15.44	7.67
Constrained by pests and diseases	0.00	0.25	1.62	5.91	−16.97	7.66
Distance to output market	−0.30***	4.74	−20.13	27.16	18.27	36.18
Distance to seed market	0.42***	4.56	−35.15	22.77	−31.37	29.78
Distance to primary school	0.06	0.99	−3.53	25.23	−29.99	35.76
Distance to extension service	−0.03***	3.08				
Access to varietal information	0.50***	4.14				
Biu LGA	−0.03	0.30	−5.63	49.80	−13.80	59.81
Kwaya Kusar LGA	−0.41***	3.55	145.15***	56.63	41.20	66.27
Intercept	−15.01***	8.44	926.27	902.22	552.27	1216.22
Model diagnosis						
$\sigma_{a}$ (adopters)			638.85***			
$\rho_{a}$ (adopters)			0.33**			
$\sigma_{n}$ (non‐adopters)					451.94***	
$\rho_{n}$ (non‐adopters)					0.36	
Wald $\chi^{2}$	50.65***					
Log pseudo‐likelihood	−88,868.76					
LR test of independent equations $\chi^{2}$	6.02**					
*N*	1094		818		276	

Notes

***, **, and * denote significance at 1%, 5%, and 10% levels, respectively.

**TABLE 4:** Full information maximum likelihood of endogenous switching regression—Soybean net revenue

Variable	Selection equation		Outcome equations			
			Adopters		Non‐adopters	
	Coefficient	std. err.	Coefficient	std. err.	Coefficient	std. err.
Male headed household	0.18	0.11	40,796.67***	5164.60	29,369.48***	7067.04
Education of HH head	0.41***	0.10	−4780.46	4459.17	−11,051.43	6928.51
Household size	−0.05***	0.01	1641.60	600.82	−274.10	727.36
Membership of association	0.21*	0.11	−1646.10	5337.60	−1754.42	7941.75
Access to credit	0.61**	0.23	6542.86	10,288.32	−31,969.87**	15,312.24
Years HH is resident in community	0.94***	0.07	−9384.98*	5423.82	−37,284.13***	8297.48
Access to off‐farm income	0.35***	0.11	6114.62	5308.92	11,626.57	7367.37
Value of HH assets	0.00	0.04	2153.65	1748.89	1159.93	2549.35
Value of farming implements	1.12***	0.17	7558.35	9247.10	13,345.27	12,615.00
Mobile phone	0.21	0.17	19,746.81	9689.46	−2516.85	10,236.91
Tropical livestock unit	0.08	0.10	2034.12	4415.64	−1144.45	6768.70
Total land cultivated	0.28***	0.08	−3331.61	3827.51	7648.02	5168.62
Use of SSP	0.67***	0.12	−22,228.82***	6212.51	10,846.73	8785.41
Use of herbicide	−0.53***	0.15	−22,004.10***	7146.62	−15,981.14	10,520.46
Constrained by low soil fertility	0.01	0.01	807.53	635.48	−524.51	878.42
Constrained by high cost of inputs	−0.02	0.01	−334.73	671.02	1901.43**	955.96
Constrained by pests and diseases	0.00	0.01	489.59	645.62	−1190.09	951.87
Distance to output market	−0.30***	0.06	−3010.26	3000.55	4795.94**	4391.37
Distance to seed market	0.42***	0.09	−2698.58	2494.82	−13,389.84***	3676.86
Distance to primary school	0.06	0.06	1496.80	2755.69	−3337.91	4432.41
Distance to extension service	−0.03***	0.01				
Access to varietal information	0.55***	0.12				
Biu LGA	−0.01	0.12	1844.21	5441.82	−12,633.07	7454.70
Kwaya Kusar LGA	−0.39	0.11	13,922.84	6261.15	616.45	7991.17
Intercept	−14.92	1.79	106,948.40	105,317.50	125,628.70	138,492.30
Model diagnosis						
$\sigma_{a}$ (adopters)			69,549.90***			
$\rho_{a}$ (adopters)			0.22			
$\sigma_{n}$ (non‐adopters)					56,961.62***	
$\rho_{n}$ (non‐adopters)					0.45**	
Wald $\chi^{2}$	139.0***					
Log pseudo‐likelihood	−14,041.305					
LR test of independent equations $\chi^{2}$	5.76**					
*N*	1094		818		276	

Note

***, **, and * denote significance at 1%, 5%, and 10% levels, respectively.

The results show that the level of education of farmers has a positive and statistically significant effect on the adoption of ISVs. This is in tandem with previous studies (e.g., Khojely et al., 2018; Manda et al., 2019; Wossen et al., 2019) that have reported a positive effect of education on improved technology adoption among rural households in Sub‐Saharan Africa. This is expected given that education improves a farmer's ability to understand the benefits of new technology, as it plays a crucial role in farmers adopting a new technology (Feder et al., 1985). Household size has a negative and statistically significant effect on the adoption of ISVs, and this is not consistent with Zheng et al. (2021) who reported that household size had a positive influence on the adoption of improved organic agricultural practices in China. This is expected as households with larger sizes are less likely to face labor constraints. However, larger families sometimes attach greater importance to non‐farming activities compared to smaller households, which may result in a negative correlation between household size and improved technology adoption (Kafle, 2010).

Differences in resource endowment such as access to credit, access to off‐farm income, and value of farm endowments have positive and statistically significant effects on the adoption of ISVs. This is in line with the economic constraint theory of adoption, which states that differences in resources such as income, land, or capital will lead to differences in the adoption of new technologies (Adesina & Zinnah, 1993). Our findings are empirically consistent with studies such as Teklewold et al. (2013) that reported, access to credit had a positive and significant effect on improved maize variety adoption in Ethiopia. Our results are also consistent with the findings of Danso‐Abbeam et al. (2017) who reported that access to off‐farm income had a positive and significant effect on the adoption of improved maize varieties in Ghana.

Our findings reveal that social capital is very important to the adoption of ISVs, which is consistent with many studies in the technology adoption and impact literature (Ali et al., 2018; Danso‐Abbeam et al., 2017; Donkor et al., 2019; Teklewold et al., 2013; Wossen et al., 2019). Social capital variables such as membership of association have a positive influence on the adoption of ISVs. This is plausible because it helps to reduce transaction costs, create collective action, and help in the diffusion of information among members in a social network (Husen et al., 2017). Consistent with our findings are studies such as Donkor et al. (2019) and Abebaw and Haile (2013) who reported that membership of association had a positive and significant impact on the adoption of improved agricultural technologies. Our findings are consistent with many studies that have reported access to varietal information, one of the instrumental variables, as being a significant determinant of the adoption of improved technologies (Abdoulaye et al., 2018; Chandio & Yuansheng, 2018; Murray et al., 2016; Wossen et al., 2019).

Total land cultivated has a positive and significant effect on the adoption of ISVs and this is logical for several reasons. Firstly, the more lands farmers have to cultivate, the greater their ability to raise capital through rent or sale to buy inputs. Secondly, land may be an indicator that a farmer is sufficiently endowed with the resources required to adopt a new technology for a sustainable period. Oyinbo et al. (2019) and Ali et al. (2018) are examples of two recent studies that have also reported the influence of cultivatable land on the adoption of improved agricultural technologies. The use of herbicides was found to have a negative and significant impact on the adoption of ISVs. This is likely because weeds which are among the major constraints to crop production in the Nigeria savannas are heavily suppressed by soybean (Menkir et al., 2020) because of its aggressive growth and ground cover. Thus, farmers that grow and invest in soybean on their plots may not see the need to invest in herbicides to control weeds.

Spatial variables such as distance to output market and distance to extension service providers are negatively correlated with the adoption of ISVs. This is expected as farmers who reside closer to extension service providers and have access to markets are more likely to have better access to information on ISVs and related technologies, which can lead to better‐informed decisions on ISV adoption. This is consistent with other empirical studies that show that distance to the agricultural extension office and markets are important in agricultural technology adoption (Kassie et al., 2015; Khojely et al., 2018; Manda et al., 2019; Shiferaw et al., 2014). Unlike these studies, distance to the seed market was found to have a positive effect on adoption and this is also plausible as farmers that can access markets further away are more likely to be exposed to new technologies than those whose seed market is limited to more localized markets.

#### 5.2.2 ESR estimates of the determinants of soybean yield and net revenue

Results for the outcome equations of yield and net revenue are shown in columns (3) to (6) of Tables 3 and 4. The estimated coefficients of the explanatory variables for the adopter and non‐adopter regimes have different signs and magnitudes for some of the variables, which indicates that the switching regression approach is preferred over a simple treatment effects model, as it captures heterogeneity between the two adoption categories (Jaleta et al., 2018; Kabunga et al., 2012; Tufa et al., 2019). Table 3 shows that household size, for example, has a positive and significant influence on yield only for adopters of ISVs. This is plausible as households with a larger size are less likely to face labor constraints. This allows such households to save on labor costs and to buy other important inputs such as fertilizers which help to increase yield (Abdulai & Huffman, 2014; Kabunga et al., 2012). Consistent with Kabunga et al. (2012) the determinants of yield for non‐adopters were found to be plot level inputs such as the use of herbicides, use of SSP fertilizer, and farming implements. These inputs are very important to the production function and are required to boost yield. Other important determinants of yield for the adopters of ISVs include access to off‐farm income, the value of household assets, mobile phone ownership, and the constraint of low soil fertility, which are positively associated with yield.

For the net revenue outcome (Table 4), gender has a positive and significant correlation with net revenue for both adopters and non‐adopters of ISVs, which suggests that MHHs are more likely to have higher net revenues. This is not surprising as Table 1 shows that the MHHs are more likely to have better access to market information and, in turn, are more likely to have better bargaining power in negotiating output price. This result is consistent with Tufa et al. (2019) who found differences in soybean income between male and female households in Malawi. Notable factors that significantly explain the net revenue of adopters include the use of complementary inputs (fertilizer, herbicides) and years HH is resident in community. The factors that significantly explain non‐adopters net revenue include access to off‐farm income, years HH is resident in community, distance to output market, distance to seed market. The results show that distance to seed market is negatively correlated with the net revenue of non‐adopters. This is plausible as non‐adopters who live far from seed markets are less likely to access the soybean varieties required for them to boost their yield and net revenue. In addition, the results show that distance to the outputs market is positively correlated with the net revenue of non‐adopters. Where transaction costs are quite low, this result is plausible because farmers can get a better price for their outputs in markets located further away from the villages—e.g., markets in urban centers. This is consistent with Kabunga et al. (2012) who reported that spatial variables such as distance to the closest market were determinants of the productivity of non‐adopters of banana tissue culture in Kenya.

The lower part of Tables 3 and 4 present the model diagnostics and estimates of the covariance terms. Table 3 shows that the parameter $\rho_{a}$, which measures the correlation between the error term of the selection equation and the outcome equations for the adopters of ISVs, is positive and significant. This indicates a negative selection bias, which implies that soybean‐producing households with lower than average yields are more likely to adopt ISVs. This negative selection is consistent with the findings of Kabunga et al. (2012) who posited that negative selection bias is not implausible, as farmers who have experienced severe problems, such as drought, pests, and diseases may be more willing to adopt varieties that can address these challenges. In Table 4, the parameter $\rho_{n}$ is positive and significant, which also implies a negative selection bias as it shows that soybean‐producing households with lower net revenues are more likely to adopt and this is consistent with the findings of Fitawek and Hendriks (2021). In addition, Tables 3 and 4 show the likelihood ratio tests for joint independence of the three equations is significant. The results indicate that the equations are dependent, which implies that if we had assumed that the equations are independent, our estimates would have been considered biased.

#### 5.2.3 Impact of ISVs on soybean yield and net revenue

Table 5 shows the yield and net revenue predictions based on the estimates of the ESR model. The ATTs in Table 5 show the change in our outcomes after accounting for selection bias arising from systematic differences in observable and unobservable characteristics between the adopters and non‐adopters. The results show that the adoption of ISVs has a positive and significant impact on soybean yield and net revenue. The estimated yield for the adopters of ISVs is on average 2399.68 kg/ha and they would have obtained an average yield of 1910.24 kg/ha if they had not adopted the ISVs. The ATT, which is the difference between the yield obtained as a result of making the decision to adopt and the decision not to adopt, is 489.44 kg/ha and this represents a yield‐increasing effect of 26%. In addition, the decision to adopt ISVs led to an average net revenue of 203,305.70 NGN/ha (USD 664) and the ISV adopters would have obtained an average net revenue of 153,697.20 NGN/ha (USD 4502) had they not adopted. The ATT of 49,608.44 NGN/ha (USD 161) represents a net revenue gain of about 32%. Our findings are consistent with many empirical studies that have reported that the use of improved crop varieties and related technologies led to a positive impact on yield and net revenue of rural households in SSA (e.g., Abdulai & Huffman, 2014; Kassie et al., 2018; Khojely et al., 2018; Manda et al., 2019; Nguezet et al., 2020; Tufa et al., 2019).

**TABLE 5:** Estimated treatment effects based on the ESR model

Outcomes	Adoption decision		ATT	% gain
	To adopt	Not to adopt		
Soybean yield	2399.68 (5.53)	1910.24 (7.31)	489.44 (9.17)***	25.62
Soybean net revenue	203,305.70 (1008.27)	153,697.20 (1246.10)	49,608.44 (1602.93)***	32.27

Note

Standard errors reported in parentheses, *** denote significance at 1% level.

### 5.3 Impact of the gender of the household head on soybean yield

Table 6 shows the impact of the gender of the household head on soybean yield for MHHs and FHHs, as calculated using the ESTER model. The result reveals that if FHHs were assigned the same returns to the observed characteristics of MHHs, their soybean yield would have reduced by 43.64 kg/ha (a 1.94% reduction), and this is significant at the 1% level. Although it is statistically significant, it would be erroneous to conclude that FHHs have an advantage in soybean yield based on their characteristics because the differences in soybean yield are too marginal in size for one to conclude that gender has a significant impact on soybean yield. Thus, the results suggest that compared to the MHHs, the FHHs are not disadvantaged in terms of yield, which is similar to the findings of Ali et al. (2016) who reported that although men had greater access to inputs and assets in rural Uganda, female‐managed plots had a net endowment advantage of 12.9%. The results also show that the base level effects of soybean yield for MHHs is 2.28% (significant to the 1% level). This indicates that the soybean yield of FHHs would have been lower by 2% if the level of resource use of FHHs would have been the same for MHHs. The results also reveal that if FHHs had the same coefficients as MHHs, their net revenue would have increased by 19.44% which is 39,126.94 NGN (USD 161), and the effect is significant at the 1% level. This implies that the net revenue of FHHs would improve significantly by about 20% if they have the same returns to the observed characteristics of MHHs, which is an indication that there is gender inequality in market access. This is because it is expected that given the substantially low differences in yield between MHHs and FHHs (with FHHs having a slightly higher yield), there should be parity in net revenue between both set of households. This disparity may be because MHHs have more social capital associated with higher membership in community organizations. This may have helped them in achieving greater market power, as they can leverage collective bargaining to attract a higher output price compared to individual bargaining.

**TABLE 6:** Gender differential in soybean yield and net revenue based on the ESTER model

Outcomes	FHHs	MHHs	Returns effect	% gain
Soybean yield for FHHs	2298.08 (13.52)	2273.47 (14.37)	24.61 (12.54)***	1.08
Soybean yield for MHHs	2335.77 (12.62)	2325.35 (13.58)	10.42 (11.62)	0.44
Level effect	−37.68 (2.15)**	−51.87 (12.22)***		
% gain	1.64	2.28		
Soybean net revenue for FHHs	172,771.20 (1247.18)	203,843.70 (1713.54)	−31,072.49 (1463.37)***	21.23
Soybean net revenue MHHs	174,952.40 (1157.30)	214,079.40 (1691.75)	−39,126.94 (1542.31)***	19.44
Level effect	−2181.26 (1696.72)	−10,235.72 (2409.79)***		
% gain	1.26	5.02		

Note

Standard errors reported in parentheses, *** and * denote significance at 1% and 10% levels, respectively.

The results also indicate that the base level effects of soybean net revenue for MHHs is about 5% and it is significant at the 1% level. This suggests that if FHHs had the same resources as MHHs, the soybean net revenue of FHHs would have been 5% higher. In general, our findings are consistent with other findings that reported income differences between male and female farmers (Gebre et al., 2021; Kassie et al., 2014, 2015; Mugisha et al., 2019; Muricho et al., 2020; Oseni et al., 2015; Paudel et al., 2020).

### 5.4 Robustness checks

To check the robustness of the ESR model estimates, we report the causal effects of ISVs using the augmented inverse‐probability weighting (AIPW) method in Table 7. Although evidence shows that the instruments that we have used in the identification of the ESR satisfy all the required conditions, there is a possibility that the model may still not be properly identified. In this regard, we complement the ESR model with the AIPW model, which only accounts for observed characteristics. The adoption of ISVs increased yield and net revenue on average by 31% and 33% respectively, compared to non‐adopters. In general, the estimates in Table 7 are consistent with those obtained using the ESR approach.

**TABLE 7:** Estimated treatment effects based on the AIPW model

Outcomes	Mean value of outcomes		ATT	% gain
	Adopters	Non‐adopters		
Soybean yield	2469.69 (22.78)	1883.40 (50.94)	586.29 (43.22)***	31.13
Soybean net Revenue	207,789.08 (2543.73)	156,687.65 (5267.32)	51,101.43 (5795.88)***	32.61

Note

Standard errors reported in parentheses, *** denotes significance at 1% level.

To properly validate the accuracy of the ESTER results, we used the Oaxaca‐Blinder decomposition method (as described in Blinder, 1973; Oaxaca, 1973). According to the Oaxaca‐Blinder decomposition method as presented in the Appendix in Table A6 and A7, the gender gap in soybean yield between MHHs and FHHs is 1.47 kg/ha for adopters and 1.35 kg/ha for non‐adopters and this value is small and not significantly different from zero. However, the FHHs had a significantly lower net revenue than MHHs, with FHHs adopters having 41,859.67 NGN/ha (USD 137) less net revenue (1% significant level) than MHHs adopters and FHHs non‐adopters having 29,916.07 NGN/ha (USD 98) less net revenue (1% significant level) than MHHs who are non‐adopters. For the adopters, the gender gap in net revenue is explained by 112.08% of the FFHs structural disadvantage (Panel B, Table A7). For non‐adopters, the result is similar, as the gap is explained by 98.03% of FFHs structural disadvantage. This differences in net revenue are due to structural disadvantages, which implies that the differences in net revenue are not due to differences in access to productive inputs (endowment effect), but due to differences in returns to these resources or to unobservable terms (structural effect) for both adopters and non‐adopters of ISVs. Both the Oaxaca‐Blinder decomposition and ESTER framework results are consistent, as they both show very little yield gap between MHHs and FHHs (Table A8). In addition, they show differences in net revenue between MHHs and FHHs and they both identify the returns effect as being the main reason for the differences between both groups and not due to endowment or level effects. The determinants of soybean yield and net revenue between MHHs and FHHs are presented in Table A4 and A5 in the appendix.

## 6 CONCLUSION AND POLICY RECOMMENDATIONS

The results of the study show that the adoption of ISVs led to a positive and significant effect on soybean yield and net revenue per hectare. In addition, the results show that while there are no substantial differences in soybean yield between MHHs and FHHs, the differences in soybean net revenue between MHHs and FHHs are quite large, with FHHs having less net revenue than their male counterparts. A plausible reason for the differential net revenue in favor of MHHs could be because MHHs have a higher social capital, which allows for more bargaining power, and better access to market. More empirical studies may help to clarify the mechanisms for the differential soybean net revenue in favor of MHHs.

To increase the adoption of ISVs, our findings show that policymakers and other development partners should strengthen their collaboration towards improving farmers’ access to soybean varietal information, which is a vital entry point for adoption. It is also important for policymakers to improve farmers’ access to improved seeds through policies that can foster community‐based seed multiplication and increased linkages to seed companies. Our results also suggest that policies that can improve farmers’ access to extension education and credit facilities, and encourage group membership can be instrumental in increasing the rate of ISV adoption in the study area. Overall, our findings show that while policymakers and their development partners can leverage ISVs to boost the yields of both MHHs and FHHs, closing the gender gap in soybean income necessitates reducing the disparity in market linkages, so that FHHs can equally have better market access. This may strengthen a win‐win outcome of ISV adoption for MHHs and FHHs. Given the slow pace of development associated with the *Boko haram* armed insurgency, among other challenges in the study area, the yield and income effects that we find can translate into welfare benefits to smallholders and generate multiplier effects in the rural economy. This implies that policy interventions geared towards stimulating the growth of the rural economy in the study area should strongly support the scaling of ISVs and related technologies.

## ENDNOTES

^1^
In our study context, we considered a household as a FHH if it is strictly a *de jure* FHH—that is, a household that is managed by a woman who is single, widowed, divorced, or separated. In many gender‐based studies, data are usually analyzed using either an inter‐household (data are disaggregated by sex of household heads) or intra‐household (data are disaggregated by sex of plot managers) approach. Although the intra‐household approach is more informative, we use the inter‐household approach, due to data limitation on the sex of plot managers. However, it is worth noting that in Nigeria and many parts of sub‐Saharan Africa, sex of the household head is a good proxy for sex of the plot manager, as reported in Kilic et al. (2015) for Malawi and Muricho et al. (2020) for northern Nigeria. This potentially works well for our study setting because the study area is in a Muslim‐dominated area of Nigeria, where decisions on plot management are often made by the household heads.

^2^
We acknowledge that accurate identification of improved varieties can be very challenging in household survey that relies on farmers’ self‐reported information, which prompted recent empirical studies to consider DNA fingerprinting (e.g., Wossen et al., 2019). While we could not carry out DNA fingerprinting due to resource constraints, we took certain measures from the outset of the study to address this. First, we consulted agronomists, extension agents, agro‐dealers and leaders of farmers’ associations before the survey to elicit the pool of local names for these varieties in the survey area to help farmers correctly report the varieties planted during the survey. We had FGDs in selected communities to further elicit local names for these varieties, the common sources of these varieties and the ability of farmers who grow them to identify the seeds. Second, we provided samples of the seeds during the survey to help farmers correctly identify the varieties planted. Third, we asked whether the seed planted is freshly purchased/sourced from agro‐dealers, out‐grower schemes, and soybean‐related projects, such as N2Africa, which are the common sources of the improved seeds.

^3^
To allay concerns about possible violation of the exclusion restriction through other means, we test the sensitivity of the estimates when deviating from the assumption that our IVs are perfectly exogenous following the bounding methods developed by Conley et al. (2012). For the sake of brevity, we do not describe these methods. For a detailed description of the estimation procedure, see Clarke and Matta (2021). As implemented by Conley et al. (2012), we provide the lower and upper bounds of the estimates by using the union of confidence intervals approach of the plausible exogenous method. Specifying a minimum (0) and maximum (0.2) value of the effect of the instrument on the dependent variable (which, by the standard assumptions should be zero), we obtained a lower bound of −193.00 kg/ha and an upper bound of 803.93kg/ha for yield while for net revenue, we obtained a lower bound of −2754.67 NGN/ha (USD 9.03) and an upper bound of 116419.17 NGN/ha (USD 381.70). These results suggest that in the presence of unobserved heterogeneities and where our instruments are not perfectly exogenous, the ESR estimates are still consistent.

^4^
Link to the exchange rate in 2017—https://www.cbn.gov.ng/rates/exrate.asp?year=2017

## 1

**TABLE A1:** Summary of sampling and sample size

LGA	No. of communities	Names of communities		Sample size
Biu	14	Filin Jirgi	Kinging	280
		Yamarkumi	Maina Hari	
		Tum	Yawi	
		Grim	Vina Dam	
		Yaulari	Tabra	
		Nzukuku	Kabura	
		Mirnga	BCG	
Hawul	14	Sakwa	Ghuma	280
		Marama	Azare	
		Fumwa	Dusu	
		Tanga Ramta	Kuburdugu	
		Kigir	Kidang	
		Hema	Yimirshika	
		Mbulatawiwi	Ngwa	
Kwaya Kusar	12	Kwaya Kusar	Midla	240
		Gashina	Peta	
		Gadam	Jalingo	
		Wandali	Yimirthalang	
		Kulthidika Nguda	Guwal	
		Kurba Gayi	Gusi	
Total	40	40		800

**TABLE A2:** Instrumental variables validation for Soybean yield

Variable	Selection equation (Probit)		Yield equation for adopters (OLS regression)		Yield equation for non‐adopters (OLS regression)	
	Coefficient	*t*‐value	Coefficient	*t*‐value	Coefficient	*t*‐value
Male headed household	0.17	1.53	−19.72	0.42	−13.28	0.23
Education of HH head	0.42***	4.41	−76.55*	1.93	−75.40	1.41
Household size	−0.05***	4.73	17.30***	3.34	2.22	0.40
Membership of association	0.24	2.07	−91.89*	1.89	−99.87	1.54
Access to credit	0.65	2.86	94.93	1.02	−136.06	1.08
Years HH resident in community	0.32	2.96	−66.90	1.79	−302.65***	7.03
Access to off‐farm income	0.00	0.02	84.78*	1.76	84.62	1.40
Value of HH assets	0.21	1.22	31.45*	1.95	−16.87	0.80
Value of farming implements	0.09	0.84	18.55	0.23	91.44	0.97
Mobile phone	0.26***	3.34	149.33*	1.68	−116.46	1.38
TLU	0.67***	5.59	21.04	0.52	−4.46	0.08
Total land cultivated	−0.54***	3.56	−23.60	0.68	48.30	1.18
Use of SSP	0.01	0.77	−8.57	0.16	84.19	1.29
Use of herbicide	−0.02	1.63	63.16	0.98	−66.88	0.80
Low soil fertility	0.00	0.32	11.19*	1.90	4.32	0.59
High cost of inputs	−0.30***	4.68	−10.03	1.63	17.02**	2.14
Pests and diseases	0.93***	12.85	1.51	0.25	−17.64**	2.22
Distance to output market	0.40***	4.33	1.47	0.05	36.28	1.02
Distance to seed market	0.54***	4.48	−81.45	2.02	−16.82	0.28
Distance to primary school	−0.03***	2.99	−4.56	0.18	−39.40	1.06
Distance to extension service	1.13***	6.65	5.24	1.01	−2.16	0.43
Access to varietal information	0.05***	4.84	−61.92	1.04	−6.07	0.10
Biu LGA	−0.02	0.17	2.00	0.04	−13.83	0.22
Kwaya Kusar LGA	−0.40***	3.47	171.89	3.06	86.76	1.44
Intercept	−14.83***	8.29	2086.44	2.49	1905.88**	1.96
N	1094		818		276	
Joint test of significance of the IVs ($\chi^{2}$)	25.70***					

Note

***, **, and * denote significance at 1%, 5%, and 10% levels, respectively.

**TABLE A3:** Instrumental variables validation for Soybean net revenue

Variable	Selection equation (Probit)		Net revenue equation for adopters (OLS regression)		Net revenue equation for non‐adopters (OLS regression)	
	Coefficient	*t*‐value	Coefficient	*t*‐value	Coefficient	*t*‐value
Male headed household	0.17	1.53	39,937.04***	7.66	27,288.62***	3.78
Education of HH head	0.42***	4.41	−6741.63	1.55	−17,331.52***	2.64
Household size	−0.05***	4.73	1921.13***	3.37	209.17	0.30
Membership of association	0.24**	2.07	−2765.70	0.52	−4874.86	0.61
Access to credit	0.65**	2.86	3814.81	0.37	−39,013.46***	2.51
Years HH resident in community	0.32***	2.96	−13,950.22***	3.40	−51,158.72***	9.66
Access to off‐farm income	0.00	0.02	4938.76	0.93	7703.95	1.04
Value of HH assets	0.21	1.22	2001.94	1.13	808.96	0.31
Value of farming implements	0.09	0.84	1487.43	0.17	−1082.75	0.09
Mobile phone	0.26***	3.34	18,094.57**	1.86	−5923.56	0.57
TLU	0.67***	5.59	1461.63	0.33	−2530.86	0.36
Total land cultivated	−0.54***	3.56	−4288.56	1.12	3946.32	0.78
Use of SSP	0.01	0.77	−24,726.78***	4.14	2665.74	0.33
Use of herbicide	−0.02	1.63	−20,634.09***	2.91	−10,023.25	0.97
Low soil fertility	0.00	0.32	854.20	1.32	−679.85	0.76
High cost of inputs	−0.30***	4.68	−271.62	0.40	2067.27***	2.11
Pests and diseases	0.93***	12.85	469.65	0.72	−1184.99	1.21
Distance to output market	0.40***	4.33	−2804.46	0.91	9318.38***	2.13
Distance to seed market	0.54***	4.48	−728.77	0.16	−19,947.49***	2.72
Distance to primary school	−0.03***	2.99	1192.98	0.43	−3817.39	0.83
Distance to extension service	1.13***	6.65	−458.33	0.81	421.74	0.69
Access to varietal information	0.05***	4.84	−5186.66	0.79	−11,223.05	1.49
Biu LGA	−0.02	0.17	1580.10	0.28	−13,325.85*	1.73
Kwaya Kusar LGA	−0.40***	3.47	15,578.38**	2.52	7467.30	1.01
Intercept	−14.83	8.29	200,106.60**	2.17	307,566.90***	2.57
N	1094		818		276	
Joint test of significance of the IVs ($\chi^{2}$)	25.70***					

Notes

***, **, and * denote significance at 1%, 5%, and 10% levels, respectively.

**TABLE A4:** Determinants of soybean yield between MHHs and FHHs in North‐East Nigeria

Variable	FHHs			MHHs		
	Coefficient	Standard error	*t*‐value	Coefficient	Standard error	*t*‐value
Adoption of ISVs	644.21***	114.78	5.61	752.03***	99.58	7.55
Education of HH head	−8.37	61.30	0.14	−140.64***	43.71	3.22
Household size	10.57	8.45	1.25	15.91***	4.65	3.42
Membership of association	−93.26	62.75	1.49	−117.56*	68.80	1.71
Access to credit	−33.10	154.43	0.21	26.24	132.06	0.20
Years HH resident in community	−26.02	77.15	0.34	127.06*	71.85	1.77
Access to off‐farm income	−5.18	23.76	0.22	62.26***	20.25	3.07
Value of HH assets	172.83	120.91	1.43	−69.52	155.73	0.45
Value of farming implements	−5.65	81.09	0.07	9.32	53.61	0.17
Mobile phone	10.32	47.60	0.22	−13.32	42.75	0.31
TLU	21.78	73.71	0.30	−19.98	81.08	0.25
Total land cultivated	60.88	80.64	0.75	103.80	102.06	1.02
Use of SSP	14.20	8.89	1.60	9.99	7.37	1.36
Use of herbicide	−9.30	10.21	0.91	−3.78	6.98	0.54
Low soil fertility	−7.58	9.46	0.80	0.98	9.36	0.11
High cost of inputs	56.35	46.86	1.20	−29.21	28.64	1.02
Pests and diseases	−47.73	88.45	0.54	−204.15***	52.22	3.91
Distance to output market	−214.20**	57.86	3.70	13.81	54.58	0.25
Distance to seed market	11.96	96.82	0.12	−109.49	69.45	1.58
Distance to primary school	19.91***	5.59	3.56	−4.97	4.74	1.05
Distance to extension service	23.43	106.75	0.22	117.07	72.73	1.61
Access to varietal information	67.12**	33.64	2.00	−71.86*	37.37	1.92
Biu LGA	−70.29	66.87	1.05	9.13	71.36	0.13
Kwaya Kusar LGA	135.45*	80.79	1.68	113.19	76.18	1.49
Intercept	1583.07	1048.28	1.51	797.14	746.78	1.07
Model diagnosis						
$R^{2}$	0.21			0.24		
*F*‐test	20.49***			22.83***		
Akaike Criterion	8304.94			8829.44		
Bayesian Criterion	8411.66			8937.90		
*N*	528			566		

Note

***, **, and * denote significance at 1%, 5%, and 10% levels, respectively.

**TABLE A5:** Determinants of soybean net revenue between MHHs and FHHs in North‐East Nigeria

Variable	FHHs			MHHs		
	Coefficient	Standard error	*t*‐value	Coefficient	Standard error	*t*‐value
Adoption of ISVs	55,727.64***	11,557.07	4.82	88,236.35***	15,082.07	5.85
Education of HH head	−1193.20	6257.04	0.19	14,325.66***	5302.57	2.70
Household size	925.07	834.38	1.11	2097.61***	627.36	3.34
Membership of association	−5193.80	6110.6	0.85	4499.63	8221.22	0.55
Access to credit	−22,087.58**	11,275.12	1.96	3743.25	16,427.79	0.23
Years HH resident in community	−187.84	6768.81	0.03	10,108.20	9974.84	1.01
Access to off‐farm income	778.89	2462.95	0.32	5083.09	2915.00	1.74
Value of HH assets	14,331.44	10,058.25	1.42	23,198.27	18,085.15	1.28
Value of farming implements	1563.37	6174.73	0.25	271.77	6547.88	0.04
Mobile phone	−2734.14	5287.27	0.52	1257.42	5499.2	0.23
TLU	−15,797.49*	8286.17	1.91	19,599.38*	11,730.28	1.67
Total land cultivated	−19,367.87**	9310.32	2.08	4293.30	9785.07	0.44
Use of SSP	1115.47	962.74	1.16	573.90	915.74	0.63
Use of herbicide	−1317.16	956.00	1.38	1241.40	791.58	1.57
Low soil fertility	−164.19	938.96	0.17	20.45	1078.26	0.02
High cost of inputs	4165.44	4569.28	0.91	4606.03	4968.75	0.93
Pests and diseases	−6543.43	8781.73	0.75	37,566.96	6769.39	5.55
Distance to output market	−19,454.65***	5153.30	3.78	4637.35	8120.07	0.57
Distance to seed market	−728.45	8001.21	0.09	11,525.57	8987.73	1.28
Distance to primary school	1662.36***	591.93	2.81	1195.79	813.11	1.47
Distance to extension service	−6150.29	9956.73	0.62	13,212.86	12,368.28	1.07
Access to varietal information	8157.95***	2879.12	2.83	6525.95	4230.20	1.54
Biu LGA	−5951.23	7810.04	0.76	1558.95	8982.60	0.17
Kwaya Kusar LGA	8648.28	6833.81	1.27	11,983.07	10,394.01	1.15
Intercept	280,268.47***	93,506.26	3.00	77,773.46	124,429.00	0.63
Model diagnosis						
$R^{2}$	0.19			0.29		
*F*‐test	16.34***			11.24***		
Akaike Criterion	13,137.49			10,119.84		
Bayesian Criterion	13,244.22			10,063.69		
*N*	528			566		

Note

***, **, and * denote significance at 1%, 5%, and 10% levels, respectively.

**TABLE A6:** Oaxaca‐Blinder model for gender gap in soybean yield

	Adopters			Non‐adopters		
A. Mean yield differential	1.47 (47.03)			1.35 (65.22)		
B. Aggregate decomposition	Endowment effect	FHHs structural disadvantage	MHHs structural advantage	Endowment effect	FHHs structural disadvantage	MHHs structural advantage
Total differential	−33.60 (26.89).	53.08 (40.86).	−18.00 (53.30).	4.61 (22.78).	−28.78 (66.02)	22.78 (66.02).
Share of differential	−2,285.71%	3610.88%	−1224.49%	341.48%	2131.85%	1687.40%
C. Detailed decomposition						
Education of HH head	−4.46 (5.84)	21.99 (59.76)	−134.84 (52.84)***	−17.7 (15.72)	−8.19 (77.96)	−204.26 (80.3)***
Household size	−4.56 (6.31)	19.99 (8.85)**	19.38 (6.35)***	−1.92 (4.95)	0.23 (8.28)	3.61 (8.19)
Membership of association	11.62 (10.6)	−151.02 **(74.97)	−71.33 (63.27)	24.54 (16.79)	−15.69 (94.24)	−236.51 (94.65)***
Access to credit	−0.41 (1.47)	73.05 (142.5)	38.85 (123.61)	−4.55 (7.23)	−111.22 (174.06)	−211.31 (194.00)
Access to off‐farm income	−5.67 (5.05)	−22.07 (71.37)	104.91 (66.76)	−9.47 (10.9)	−0.8 (84.21)	159.27 (88.95)*
Value of HH assets	−9.32 (7.77)	6.10 (22.43)	69.20 (24.02)***	−5.84 (10.9)	−70.72 (30.09)**	63.97 (31.65)**
Mobile phone	−3.77 (14.88)	182.66 (105.91)*	43.64 (171.89)	42.76 (25.62)	1.14 (103.16)	−320.63 (164.57)**
TLU	1.01 (2.73)	9.33 (57.1)	22.99 (58.43)	−4.52 (6.94)	4.20 (81.87)	−66.21 (80.78)
Total land cultivated	17.70 (14.00)	16.00 (51.44)	−61.3 (47.37)	−32.92 (20.24)	26.19 (59.08)	111.7 (60.13)*
Use of SSP	0.29 (1.30)	−22.13 (79.58)	−18.25 (73.57)	1.29 (4.01)	144.49 (94.28)	56.04 (93.85)
Use of herbicide	−3.97 (4.31)	81.72 (105.52)	89.03 (82.25)	0.6 (5.52)	−106.99 (114.28)	13.84 (126.9)
Low soil fertility	−2.47 (3.58)	17.78 (9.00)**	11.29 (7.86)	−1.43 (4.91)	11.16 (11.19)	9.56 (10.38)
High cost of inputs	−0.25 (1.66)	−18.37 (9.44)**	−6.10 (8.05)	0.07 (0.87)	24.32 (12.24)**	1.42 (12.37)
Pests and diseases	−0.15 (2.8)	3.28 (8.61)	0.43 (8.19)	5.78 (10.21)	−34.95 (11.41)***	6.82 (11.51)
Distance to output market	−2.81 (4.06)	88.76 (42.03)**	−56.67 (37.81)	−2.00 (6.07)	32.46 (54.27)	17.68 (49.72)
Years HH resident in community	−24.42 (10.03)***	98.20 (63.44)	−157.07 (46.22)***	6.11 (33.27)	−312.16 (60.94)	−381.15 (66.27)***
Distance to seed market	−2.66 (4.63)	−330.57 (63.52)***	40.67 (52.71)	1.39 (7.06)	3.85 (86.08)	−18.6 (87.62)
Access to varietal information	−7.39 (5.68)	−49.10 (100.85)	−124.89 (74.84)	−0.69 (6.62)	79.58 (93.94)	−108.82 (97.48)
Distance to extension service	4.11 (5.86)	37.05 (8.64)***	−9.14 (6.46)	4.37 (9.93)	−4.6 (7.42)	−3.96 (6.90)
Value of farming implements	1.70 (2.85)	−23.82 (126.41)	69.56 (100.23)	−2.76 (7.29)	41.81 (130.25)	141.45 (140.68)
Distance to primary school	−1.53 (5.19)	72.63 (37.35)**	−80.79 (34.17)**	1.29 (7.69)	−22.68 (51.9)	−77.85 (58.73)
Biu LGA	0.39 (1.41)	−12.34 (77.37)	21.31 (67.55)	0.26 (5.76)	−68.15 (89.32)	−4.44 (98.9)
Kwaya Kusar LGA	3.43 (4.09)	203.31 (82.72)***	117.14 (78.89)	−0.06 (2.18)	112.12 (87.28)	36.56 (87.54)
Intercept	−33.6 (26.89)	2020.77 (1362.91)	1770.62 (1051.93)***	4.61 (53.47)	2721.88 (1334.08)**	1274.82 (1474.63)
Observations	818	281	437	276	147	129

Notes

Standard errors reported in parentheses, ***, **, and * denote significance at 1%, 5%, and 10% levels, respectively.

**TABLE A7:** Oaxaca‐Blinder model for gender gap in soybean net revenue

	Adopters			Non‐adopters		
A. Mean yield differential	−41,859.67 (5111.26)***			−29,916.07 (8640.32)***		
B. Aggregate decomposition	Endowment effect	FHHs structural disadvantage	MHHs structural advantage	Endowment effect	FHHs structural disadvantage	MHHs structural advantage
Total differential	−6737.07 ** (3488.94)	11,794.84 (4736.66)***	−46,917.45 (5668.34)***	−5672.93 (8561.95)	5083.53 (7686.18)	−29,326.67 (8050.98)***
Share of differential	16.09%	112.08%	−28.18%	18.96%	98.03%	−16.99%
C. Detailed decomposition						
Education of HH head	−423.81 (570.86)	4608.13 (5352.45)	−12,811.9 (6485.76)**	−3329.34 (2788.32)	−9975.34 (9289.01)	−38,411.47 (9963.68)***
Household size	−520.51 (722.52)	1886.83 (792.91)**	2211.14 (779.22)**	−528.27 (846.58)	−672.06 (986.22)	996.17 (1015.75)
Membership of association	428.15 (1268.94)	−6752.86 (6714.55)	−2627.15 (7766.34)	−214.9 (1224.68)	832.27 (11,229.66)	2070.72 (11,744.56)
Access to credit	−22.37 (164.54)	−9411.48 (12,762.55)	2116.65 (15,173.55)	−912.68 (1293.48)	−43,956.93 (20,740.56)**	−42,419.45 (24,073.11)*
Access to off‐farm income	−336.48 (489.76)	−821.14 (6392.5)	6228.90 (8194.46)	−831.46 (1063.49)	560.50 (10,034.71)	13,977.86 (11,037.51)
Value of HH assets	−600.57 (604.31)	901.83 (2009.06)	4459.25 (2948.64)	−621.25 (1174.76)	−1848.49 (3585.40)	6809.31 (3927.24)*
Mobile phone	−3436.44 (1971.33)	6172.56 (9485.23)	39,769.02 (21,099.45)*	3949.66 (2984.39)	3318.21 (12,291.80)	−29,615.41 (20,420.61)
TLU	99.6 (328.16)	549.67 (5114.12)	2265.64 (7171.88)	−1832.54 (1843.44)	9293.09 (9755.76)	−26,865.51 (10,023.31)***
Total land cultivated	1609.05 (1700.95)	−2006.79 (4607.46)	−5570.78 (5814.72)	−4122.55 (2517.17)	−3000.87 (7039.68)	13,988.91 (7461.85)*
Use of SSP	486.77 (947.74)	−2071.18 (7127.19)***	−30,422.76 (9030.57)***	427.36 (1154.26)	−230.59 (11,234.36)	18,630.04 (11,645.58)
Use of herbicide	442.57 (515.87)	−3508.80 (9450.38)***	−9916.07 (10,096.28)	−557.03 (895.21)	−10,922.74 (13,617.13)	−12,897.46 (15,746.49)
Low soil fertility	−286.43 (419.90)	1529.51 (805.94)*	1306.88 (964.85)	−22.99 (206.46)	−40.35 (1333.82)	153.96 (1288.13)
High cost of inputs	48.39 (320.69)	−2444.25 (845.06)***	1190.99 (988.70)	−23.37 (229.92)	2475.81 (1458.59)*	−485.89 (1534.68)
Pests and diseases	−42.36 (345.42)	1003.03 (771.15)	123.86 (1005.29)	406.12 (1228.95)	−1626.08 (1359.28)	479.29 (1428.83)
Distance to output market	−373.50 (530.98)	5378.33 (3764.41)	−7519.95 (4641.04)	−116.21 (710.10)	14,728.36 (6466.23)**	1028.16 (6169.92)
Years HH resident in community	−414.51 (1472.32)***	13,466.36 (5682.07)**	−26,459.44 (5673.59)***	1290.15 (7023.58)	−40,226.63 (7261.26)***	−80,490.03 (8223.42)***
Distance to seed market	−848.06 (1069.26)	−2886.15 (5689.17)***	12,949.05 (6469.91)*	1480.64 (2873.13)	−23,841.18 (10,257.61)*	−19,757.09 (10,872.59)*
Access to varietal information	−614.56 (618.65)	910.79 (9032.62)	−10,385.87 (9186.19)	−173.61 (1662.58)	−5963.17 (11,193.20)	−27,435.06 (12,096.00)**
Distance to extension service	1089.70 (1395.86)	3793.52 (773.85)***	−2424.97 (792.91)***	−208.57 (993.12)	313.17 (884.34)	188.80 (855.78)
Value of farming implements	328.91 (413.32)	−10,597.87 (11,322.07)	13,484.29 (12,303.85)	−530.53 (1344.27)	−15,091.85 (15,520.09)	27,225.26 (17,456.80)
Distance to primary school	−134.36 (460.33)	8480.27 (3344.97)**	−7114.14 (4194.75)*	181.77 (1084.68)	−980.75 (6184.72)	−11,007.27 (7288.12)
Biu LGA	57.66 (182.51)	5477.69 (6929.60)	3176.56 (8292.03)	635.75 (948.54)	−13,603.01 (10,643.32)	−10,919.97 (12,272.44)
Kwaya Kusar LGA	426.11 (506.14)	15,945.94 (7408.27)**	14,562.11 (9684.08)	−19.08 (651.25)	9005.07 (10,400.33)	10,963.67 (10,862.60)
Intercept	−6737.07 (3488.94)	23,4027.10 (122,066.30)*	123,035.80 (129,124.70)	−5672.93 (8561.95)	431,383.80 (158,966.20)***	117,794.80 (182,982.40)
Observations	818	281	437	276	147	129

Note

Standard errors reported in parentheses, ***, **, and * denote significance at 1%, 5%, and 10% levels, respectively.

**TABLE A8:** Estimating IV bounds with plausibly exogenous estimation

Outcomes	Plausible exogenous estimation (UCI)	
	Lower bound	Upper bound
Soybean yield	−193.00	803.93
Soybean net revenue	−2754.67	116,419.17
